# Cord Blood Mononuclear Cells Have a Potential to Produce NK Cells Using IL2Rg Cytokines

**DOI:** 10.15171/apb.2016.02

**Published:** 2016-03-17

**Authors:** Nahid Khaziri, Momeneh Mohammadi, Zeinab Aliyari, Jafar Soleimani Rad, Hamid Tayefi Nasrabadi, Hojjatollah Nozad Charoudeh

**Affiliations:** ^1^ Stem Cell Research Center, Tabriz University of Medical Sciences, Tabriz, Iran.; ^2^ Tissue Engineering Group, Novin School of Advanced Research Sciences, Tabriz University of Medical Sciences, Tabriz, Iran.

**Keywords:** Cord blood, Mononclear cells, NK cell, IL2rg cytokines

## Abstract

***Purpose:*** Although bone marrow represents the main site for NK cell development and also distinct thymic-dependentNK cell pathway was identified, the cytokines effect on the NK cell generation from cord blood is unclear. Studies were identified the role of cytokines in the regulation of bone marrow and thymic NK cells. Previous studies reported that IL15 are critical for bone marrow dependent and IL7 is important for thymic NK cells. It is remain unclear the cytokines influence on the expantion of NK cells in cord blood mononuclear cells.

***Methods:*** We evaluated cultured cord blood mononuclear cells suplememnted with combinations of cytokines using FACS in distinct time points. In this study, we presented the role of IL2, IL7 and IL15 as members of the common gamma receptor -chain (Il2rg) on the expansion NK cells from cord blood cells.

***Results:*** By investigating cord blood mononuclear cells in vitro , we demonstrated that IL2 and IL15 are important for expansion of NK cells. IL2 in comparision with IL15 has more influences in NK cell expansion. In contrast IL-7 is dispensable for NK cell generation in cord blood.

***Conclusion:*** Thus,IL-2Rg cytokines play complementary roles and are indispensable for homeostasis of NK cell development in cord blood. Probably these cytokines could help to use NK beneficials in engrafment of transplanted cells and Anti tumor activity of NK cells.

## Introduction

Natural Killer (NK) cells are large granular lymphocytes as a major compartmment of innate immunity, represent as a third lymphoid lineage.^1,2^ They destroy stressed cells, tumor cells and virus infected cells without any immunization and pre activation.^3^ NK cells are well-defined phenotypically as CD56+CD3– lymphocyte.^4^ NK cells express natural cytotoxicity receptors (NKP30, NKP44,NKP46). The activating receptor NKp46 is expressed on all human NK cells and rarely on T cells.^5,6^ It is one of the best markers for NK cells characterization.^7^

NK cell transferring is a best strategy for cancer immunotherapy which Killer immunoglobulin like receptors (KIRs) as inhibitory receptors for HLA class I play an essential role in the anti-leukemic effects of allogeneic NK cell transfer.^8^ In addition, alloreactive NK cells eliminate residual host dendritic cells, thus prevent graft-versus-host-disease.^8^ Because NK cells are a fraction of peripheral blood mononuclear cells, the development of methods to produce large numbers of functional NK cells could be useful to optimize NK-based therapies.

Although significant progress has been made in finding the cytokine regulation and generation of NK cells from different sources like bone marrow, spleen and thymus, the effect of cytokines and function of generated NK cells from cord blood cells remain largely unknown. The molecular and cellular mechanisms that regulate NK cell development and differentiation of these cells into effector cells have been partially charactrized.^2^ For regulation and development of NK cells several cytokines from commom gama chain family are essential , and IL-15 is the dominant common g-chain cytokine for conventional NK cell generation, survival and expantion from both mouse and human NK progenitors with ability of cytotoxic function.^9-12^ In contrast, the common g-chain cytokine IL7 is necessary for generation of thymic NK cell development with acquision of secrete inflamatory cytokines, although it is crucial for the development of B and T lymphocytes.^13-17^ This NK cell ability developed through transcription factor–mediated expression of cytokine receptor genes, and gain the capacity to respond to environmental factors.^18,19^ IL-2 is the firstly identified member of IL2rg family, and its gene was originally cloned on the basis of the T-cell growth factor activity of this cytokine.^20,21^ Besides its T cell growth factor activity, IL-2 up-regulates NK cell proliferation and function, induces lymphokine-activated killer (LAK) activity, and also mediates activated B cell proliferation and Ig production.^21,22^

Umbilical cord blood is an easy access sources to use in transplantation and immunotherapy. Probably NK cell could be geenerate from cord blood cells. It is critical to understand the influence of common comma chain cytokines like IL2, IL7 and Il15 on NK cell expansion from cord blood mononuclear cells. In this study, we evaluated the effect of IL2, IL7 and IL15 on the generation of NK cells from cord blood mononuclear cells. We stablished cytokine condition for in vitro expansion of NK cells from cord blood mononuclear cells.

## Materials and Methods

### Cell isolation and culture condition 

Cord blood samples, collected from full-term normal deliveries. All samples were diluted 2:1 with phosphate-buffered saline (PBS- SIGMA). Mononuclear cells were isolated by centrifugation on Ficoll-paque (GE healthcare, 1.078 g/ml) at 850 gm for 25 minutes. The mononuclear cells were collected, washed twice in RPMI 1640 (Gibco) supplemented with 10% FBS (Gibco).

The 10^5^ cord blood mononuclear cells were seeded in 96-well plates in 200 µL of RPMI1640 (Gibco) including 20% fetal bovine serum (FBS; Gibco), 1% penicillin/streptomycin (Gibco), in addition supplemented with cytokines including SCF, Flt3 ligand, IL- 7, IL-15, and IL-2 . All cytokines purchased from PeproTech Company (Germany) and have used with a final concentration 40 ng/ml. Cells were cultured at 37°C for 21 days.

### Monoclonal antibodies and Flow Cytometry analysis: 

Briefly, cells were incubated with Anti-NKp46-PE (BD,biosience), anti- CD3 (UCHT1; R&D) for 20 minutes at 4°C. Propidium iodide (1.0 mg/mL; Invitrogen) have used to exclude dead cells. Harvested cells analyzed at days 7, 14 and 21 using BD caliber (BD ebiosciences), and FACS plots prepared using following 2 software (Perttu Terho, version: 2.5.1.).

### Statistical analyses

Data was presented using mean (Standard Deviation: SD). The differences between groups were assessed using the Student *t* tests for comparing two groups and one-way analysis of variance (ANOVA) for comparing more than two groups. In all analyses, P < 0.05 was considered to be statistically significant. The analysis performed by Graph Pad Prism software (version: 5.04).

Experimental Ethical matters have been approved by Ethical committee of Tabriz University of medical Sciences.

## Results

### Mononuclear cells of umbilical cord blood can be efficiently expanded into NK Cells 

**Figure 1:**
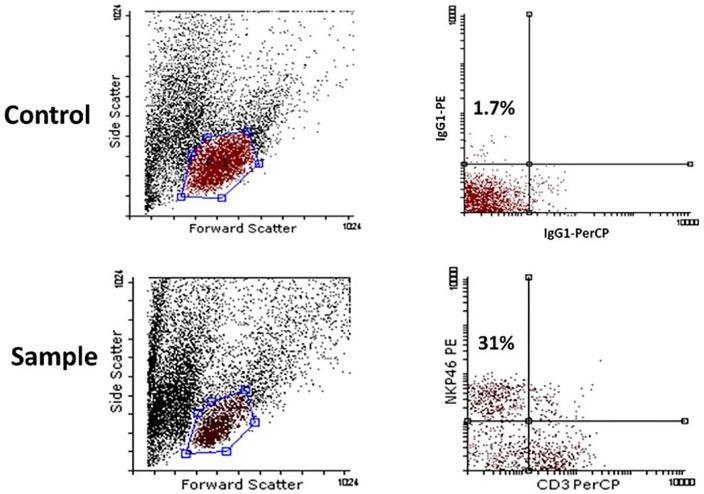
Representative flowcytometry profile of NK cell derived from 105cultured cord blood mononuclear cells. Harvested cells evaluated for NK cell (NKP46+ CD3- cells) by FACS in day 7, 14 and 21.

The IL2Rg cytokines(common cytokine receptor γ chain) including Il2, IL7 and IL15 have been shown previously to be important for NK cells development in bone marrow. Here, we evaluated IL2, IL7 and IL15 influence on NK cell generation from cord blood mononuclear cells in different combinations. The 1x10^5^ cord blood mononuclear cells were cultured for 21 days in presence of combinations of IL2, IL7 and IL15. The SCF and Flt3 were supplemented in to all groups. Harvested cells evaluated by FACS at distinct time points as shown in Figure 1.

**Figure 2:**
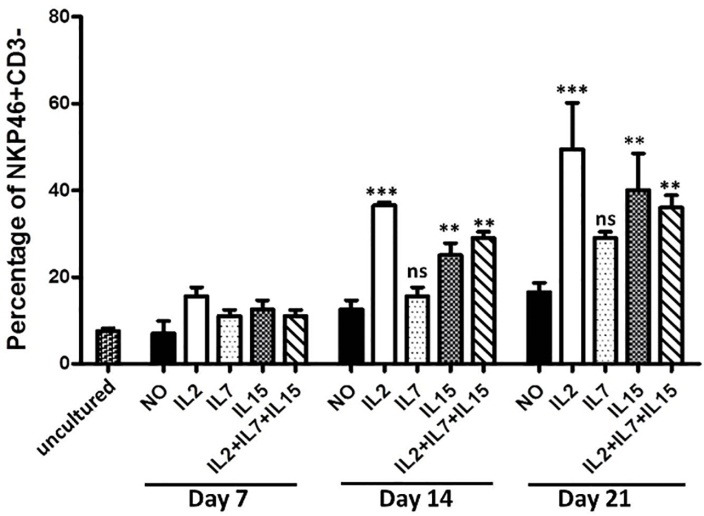
Percentage of NK cells derived from cultured cord blood mononuclear cells in presence of different cytokine conditions. Represented mean (SD) percentage of NKP46+ CD3- cells of cultured cord blood mononuclear cells in different time points and in presence of different combination of cytokines. SCF+FLt3 were included in all groups. NO= No IL2, IL7, IL15 **: P<0.05, ***: P<0.01, ns: non-significant.

NK cells increased from day 7 to day 21 in all groups, although it was not significant for IL7 groups. In presence of the IL2, the Percentage of NKP46+ CD3- cells increased from day 7 (approximately %17) to day 21 (around %50) (Figure 2).

However, the Percentage of NK cells in presences of IL15 increased from nearly 14 percent in day 7 to around 40 percent in day 21. In combination of all cytokines together in culture, there was a significant trend in the percentage of NK cells, although it was slightly lower than in presence of IL2 and IL15alone in day 21. This probably because of sharing signaling pathways in common gama chain receptors (Figure 2). In day 14 and day 21, the percentage of NKp46+CD3- cells expansion in presence of IL2 was around 10 to 15 percent more than IL15.

## Discussion

Natural Killer cell development is controlled by several cytokines like IL2, IL7 and IL15 which known as common cytokine-receptor gamma-chain.

The present study has shown the dominant role of IL2 and IL15 on the expansion of NK cells from cord blood mononuclear cells. In particular NK cell expansion was influenced by IL2 more than IL15.

Common gamma chain cytokines are soluble mediators of intercellular signals and play a critical role in the regulation and activation of adaptive and innate immunity. Common gamma chain family has a functional redundancy in the homeostasis of the lymphoid system, but each member has also its own specific functions.^23^ Two members of common gamma chain family, IL-2 and IL-15 can bind with high affinity to IL-2Rα (CD25) or IL15-Rα, respectively and of IL-2Rβ (CD122) and gamma chains.^24,25^

IL-2 play through two types of receptors: the high affinity receptor formed by IL-2Rα, IL-2Rβ and gamma chain, and an intermediate affinity receptor formed by the IL-2Rβ and gamma chain. While the high affinity receptor is expressed on activated T and NK cells, the intermediate receptor is constitutively expressed on NK cells^26^ which can directly respond to high concentrations of IL-2. IL-15 and its specific receptor IL-15Rα are essential for differentiation of immature NK cells, survival and proliferation of NK cells.^9,12,16^ IL-15Rα alone has a high affinity for IL-15 and is expressed in several lymphoid and non-lymphoid cells.^27,28^ Although recent observations indicate that IL-2 or IL-15-activated NK cells display a different sensitivity in reaction to target cells.

For future investigations, it is critical to characterize NK cells derived from CD34 positive cord blood cells. In particular, regards to cell surface markers like NCRs (NKP30, NKP44 and NKP46), KIRs (inhibitory and Activatory receptors) and also functional studies whether they are cytotoxic or cytokine producer are necessary.

In contrast, the common g-chain cytokine IL-7 seems to be dispensable for NK cell development, although it is crucial for the development of adaptive B lymphocytes and T lymphocytes.^13-16^

However it has been identified that a thymic pathway of NK cell development characterized by expression of GATA-3 and CD127.^17^ Also, the capacity of IL-7 to modulate the proliferation and function of CD127+ NK cells in both mice and humans still remain unclear. Whether different types of NK cells are producible from CD34 positive cord blood mononuclear cells need further investigations.

## Conclusion

IL2 and IL15 have dominant role on the expansion of NK cells from cord blood mononuclear cells. Especially NK cell expansion was effected by IL2 more than IL15.

## Acknowledgments

The authors thank Nazli Saeedi for helpful flowcytometry at Research Center for Pharmaceutical Nanotechnology in Tabriz University of Medical Sciences. This work has been approved by Novin School of Advanced Medical Sciences and financially supported by Research Council of Tabriz University of Medical Sciences.

## Ethical Issues

Not applicable.

## Conflict of Interest

The authors report no conflicts of interest.
